# Supplementary material for: Comprehensive characterization of mitochondrial bioenergetics at different larval stages reveals novel insights about the developmental metabolism of Caenorhabditis elegans
Source: PLoS One. 2024 Nov 26;19(11):e0306849. doi: 10.1371/journal.pone.0306849 (PMC11593755; doi:10.1371/journal.pone.0306849)
Supplement: S2 File — Please note that this protocol includes information for adult lifestages as well and larval lifestages, and information about using a 96- instead of 24-well Seahorse instrument. (DOCX) [file pone.0306849.s002.docx]

**XFe24 orXFe96 Seahorse Extracellular Flux Analyzer Preparation**

Based on initial XFe24 protocol from 2019-12-16

Most recent update 2024-08-24 by Joel Meyer

Contact [joel.meyer@duke.edu](mailto:joel.meyer@duke.edu) for updates

For original protocol, validation, and other things we tried, please see:

Luz et al., 2015 Current Protocols:

<https://www.ncbi.nlm.nih.gov/pmc/articles/PMC4632645/pdf/nihms731144.pdf>

Luz et al., 2015 PLOS ONE:

<https://pubmed.ncbi.nlm.nih.gov/26106885/>

**Purpose**

Assessing oxygen consumption rate of *C. elegans* as a measure of mitochondrial function. Notes: non-mitochondrial OCR is also measured and may be interesting. In our experience, no extracellular acidification can be detected using worms, unlike the case with cell culture.

**Reagents required to run assay (does not include standard nematode culture materials)**

- Drugs and liquid for *C. elegans*
  - DMSO (CAS 67-68-5)
  - K-medium (Williams and Dusenberry, 1988)
    - K-medium recipe provided at the end
  - “EPA Water” (EPA, 2002)
    - EPA water recipe provided at the end
  - FCCP (CAS 370-86-5)
  - DCCD (CAS 538-75-0)
  - Sodium Azide (CAS 26628-22-8)
- Seahorse parts (listed by Agilent part number)
  - 24 well XFe24 (note that these parts are not interchangeable with XF24 parts):
    - Calibration plate (sensor cartridge) and cell culture microplates sold as FluxPack containing both: 102340-100
    - Calibration solution (included in FluxPack)

**Note: lab has previously attempted use of Cell-Tak and Eyelet plates to adhere/ensure worms remain in microchamber. The eyelet plates did not work (worms slip through) and Cell-Tak did not improve outcomes (they did not adhere for long, even after centrifugation onto Cell-Tak).

- - 96 well XFe96 (parts are described on the Seahorse website as interchangeable with the XFPro as of July 2024):
    - Calibration plate (sensor cartridge) and cell culture microplates sold as FluxPack containing both: 103792-100
    - Calibration solution (included in FluxPack)
    - In our experience, the 96-well plate gives tighter replicates (wells) and less-noisy data (within wells) than the 24-well. We suspect that this is because worms are less likely to slip out of the microchamber during re-oxygenation, but do not know this for sure.

**A note regarding experimental planning and statistical analysis**

We have spent a lot of time discussing what defines biological and technical replicates when utilizing this assay. Ideally, biological replicates in this case should be defined based on exposures. I.e., exposures that occurred in one container (plate, flask, etc) are one biological replicate, and technical replicates are the wells derived from that single container on the seahorse plate. If intending to use more than one biological replicate per seahorse plate, conduct the exposures in separate containers. In any case, for publication, all experiments should include minimally two or three replicates separated in time—i.e., even if you perform multiple separate exposures and test them in separate wells, but you do them all at exactly the same time, don’t trust the results without repeating.

**Equipment storage and use**

For this protocol, the heating plate is turned off and equipment is deliberately stored in a room that is 20 C or cooler to minimize temperature increases and thermal stress to the worms during the run. Do NOT turn the heating plate on, and ensure it is OFF when the machine is turned on. It takes a substantial amount of time to cool, which can be accelerated by placing ice packs on the plate tray.

For the 96 well seahorse in our laboratory, the custom-built water-cooling system can be turned on after the machine is turned on to maintain the temperature. Additionally, aiming a small fan around the machine’s venting area to ensure rapid air flow helps maintain the reduced temperature. Temperature is recorded for each run and has historically ranged from 22-25 C when stored in the cooler (18-20 C) room, or when the water system and fan are employed. According to Koopman et al. 2016 (<https://pubmed.ncbi.nlm.nih.gov/27583642/>), there is no detectable difference in OCR between 20 and 25 C.

**Protocol**

**Egg Preparation (see separate Egg Prep protocol) and Synchronization of Worm Population**

1. Prepare a mostly day-2 adult plate. There are a number of ways to do this—for example, from a previously synchronized population, or you can grow worms from a timed egg lay (eg eggs laid over 24 h), or by washing away the larvae and adults from a mixed population plate full of eggs and waiting ~96h. Day 2 adults obtained by any of these methods should give ~10 eggs each when egg prepped as described following.
2. Wash the plate(s) of worms obtained in step 1 with K-med solution
3. Transfer the worms into a 15mL tube
4. Repeat steps 2 and 3 for the desired amount of worms
5. Settle the worms by gravity, aspirate the supernatant, add ~5 ml K-med
6. Repeat step 5 at least one more time (*to make sure there is no carryover of eggs already laid from the plate*), not adding the 5 mL of K-med the last time.
7. Add 3.5 mL of K-med (first! don’t add concentrated bleach or NaOH directly to worms), 1000 µL of sodium hypochlorite (5%) and 500 µL of 5N NaOH to the 15 mL tube and worm pellet. If there are too many worms (>~2000), they will need to be separated into multiple tubes for a successful bleach. For additional detail and other options please see our detailed egg prep protocol.
8. Place the tube in the orbital shaker inside the 20°C incubator for 6-10 minutes
9. Every 2-3 minutes, remove the tube from the shaker to briefly vortex (~10 s) and quickly observe the fragmentation
10. When worms are disintegrated (at 10 minutes or prior; note rate may vary by 2-3 minutes based on worm strain and ratio of egg prep solution to worms), complete to 15mL with K-med to stop the reaction
11. Centrifuge the eggs (swinging bucket centrifuge ideally) at 2200g for 2 minutes – look for small pellet
12. Aspirate the supernatant carefully to not disturb the pellet
13. Re-suspend the eggs in 15 ml K-med
14. Repeat steps 11 and 12 (*re-suspend to have at least 50-100 embryos/10* µL)
15. Pipette 10µL as a row of 3-5 tiny droplets of the egg suspension onto a glass slide
16. Using a light microscope count the number of eggs in each 10uL droplet
17. Repeat steps 15 and 16 (or 2x if the initial two counts are >20% apart)
18. Calculate the average of number of eggs in 10µL
19. Estimate the number of eggs in the total solution
20. Add eggs to OP50-seeded agar plates *(#worms per plate will depend on the stage at which you will harvest the worms)*
21. Plan synchronization taking into consideration time needed for nematodes to reach desired larval stage:

Table 1: Time endpoints for incubation at 20°C by N2 developmental stage

| Larval Stage | mid-L1 | mid-L2 | mid-L3 | late-L3 | mid-L4 |
| --- | --- | --- | --- | --- | --- |
| Hours since hypochlorite treatment: | 21 | 34 | 43 | 46 | 53 |

**Seahorse Plate Preparation and Run**

1. At least 4 hours before, hydrate the sensor cartridge overnight using the XF Calibrant solution at room temperature in each well. Cartridges can be hydrated a maximum of 4 days in advance. Store at room temperature or in 20 C incubator until use, with lid sealed with tape.
   1. 24 well: 1 mL/well Calibrant

96 well: 100 µL/well Calibrant

1. To set up the Seahorse software, create a new experiment design on the software, and indicate the “Injection Strategy” by identifying the drugs (Sodium azide, FCCP, DCCD), final concentrations, solvent and percent solvent used in each port. FCCP and DCCD cannot be injected into the same well. Wells should be set up such that FCCP **OR** DCCD and sodium azide are injected into each well.
   1. Protocols can be saved and loaded in the future within the software to avoid repeating this process each time. After fully designing protocol, click “save as”, and ensure the file saves as a protocol format.
2. Use “Create Groups” feature to indicate drug injection concentration groups.
3. Use the “Plate Map” feature to create groups of treatments.
4. Set up 8 cycles of “Basal Measurement Cycles,” 1 mix cycle to oxygenate the micro-chamber, 3 wait cycles to allow the worms to settle, and a measuring cycle to 3 minutes (Luz et al. 2015). Set up 14 measurement cycles for FCCP response, 14 cycles for DCCD response and 6 measurement cycles of NaAz response. (In the 24-well plate format, we usually run FCCP and DCCD injections on entirely separate plates, in which case we often ran 8 cycles for FCCP and 14 for DCCD (Luz et al., 2015 Current Protocols))
   1. The 96 well offers an acclimation step. KM and JH do not include the acclimation step for worms; we think it is not clear that any benefit of this step outweighs the time the worms spend in the wells.
5. Prepare stocks of the ETC inhibitors, appropriate for larval stage and plate format. Updated and optimized concentrations for each larval stage from Mello et al. “Comprehensive characterization of mitochondrial bioenergetics at different larval stages reveals novel insights about the developmental metabolism of *Caenorhabditis elegans*” are presented below, as are separately-optimized young adult and 8-day adult concentrations.
   1. 24 well:
      1. Note: Initial well volume is 525 µL, each injection will be 75 µL
      2. FCCP: final well concentration 25 µM in 2% DMSO
         1. Stock solution:
            1. Prepare 1.25 mM in 100% DMSO FCCP (254.17 g/mol): 0.31771 mg/mL
            2. This stock can be frozen and stored at -80 C. Do not store longer than 90 days
         2. Prepare injection port mix of 200 µM FCCP in 16% DMSO
            1. 192 µL 1.25 mM stock in 100% DMSO + 1008 µL EPA Water (is sufficient for 16 wells)
      3. DCCD: final well concentration 40 µM in 2% DMSO:
         1. Stock solution:
            1. 10 mM DCCD (206.33 g/mol) in 100% DMSO: 20.633 mg/mL
            2. KM recommends only using fresh DCCD. This stock can be frozen and stored at -80 for no more than 30 days, but 80 µM (final in-well concentration) is likely a safer choice if using frozen stocks, whereas 40 µM should be fine if using freshly-made stocks.
         2. Prepare injection port mix of 320 µM in 16% DMSO:
            1. 38.4 µL 10 mM stock in 100% DMSO + 153.6 µL DMSO + 1008 EPA water
            2. **The final well concentration is 2% DMS; this can be modified to 1% DMSO in some cases. See note at end of protocol.
      4. Sodium Azide: Final well concentration of 10 mM in H_2_O:
         1. Prepare injection port mix of 90 mM Sodium Azide (65 g/mol) in H_2_O
         2. *Need to prep for 9X dilution since it is added after other drugs (90 mM stock), only use 8X dilution if injected alone
         3. 5.85 mg/mL in EPA water for 60 mM
   2. 96 well:
      1. Note: Initial well volume is 100 µL. Each injection is 25 µL.
      2. FCCP: final well concentration 25 µM in 2% DMSO
         1. Stock solution:
            1. Prepare 1.25 mM in 100% DMSO FCCP (254.17 g/mol): 0.31771 mg/mL
            2. This stock can be frozen and stored at -80 C. Do not store longer than 90 days
         2. Prepare injection port mix of 125 µM FCCP in 10% DMSO
            1. 120 µL 1.25 mM stock in 100% DMSO + 1080 µL EPA Water (is sufficient for 48 wells)
      3. DCCD: final well concentration 40 µM in 2% DMSO:
         1. Stock solution:
            1. 10 mM DCCD (206.33 g/mol) in 100% DMSO: 20.633 mg/mL
            2. KM recommends only using fresh DCCD. This stock can be frozen and stored at -80 for no more than 30 days, but 80 µM (final in-well concentration) is likely a safer choice if using frozen stocks, whereas 40 µM should be fine if using freshly-made stocks.
         2. Prepare injection port mix of 200 µM in 10% DMSO:
            1. 24 µL 10 mM stock in 100% DMSO + 96 µL DMSO + 1080 EPA water (sufficient for 48 wells)
            2. **The final well concentration is 2% DMSO; this may be modified to 1% DMSO in some cases. See note at end of protocol
      4. Sodium Azide: Final well concentration of 10 mM in H_2_O:
         1. Prepare injection port mix of 60 mM sodium azide (65 g/mol) in H_2_O
         2. *Need to prep for 6X dilution since it is added after other drugs (60 mM stock), only use 5X dilution if injected alone
         3. 3.90 mg/mL in EPA water for 60 mM

c. Optimized drug concentrations (final, in well) for other larval stages (note the day 8 (post-hatch) values may need to be re-optimized for much older worms):

|  | L1 | L2 | mid-L3 | late-L3 | L4 | young adult | 8-day adult |
| --- | --- | --- | --- | --- | --- | --- | --- |
| DCCD (µM) | 40 | 40 | 40 | 40 | 80 (40 may work if made fresh; not tested) | 80, 40 if made fresh worked for KM | 80, 40 if made fresh worked for KM |
| FCCP (µM) | 25 | 25 | 25 | 25 | 25 | 25 | 25 |
| Sodium Azide mM | 10 | 10 | 10 | 10 | 10 | 10 | 10 |

1. Transfer each ETC inhibitor into the appropriate injection port. FCCP or DCCD will be loaded into Port A, and sodium azide will be loaded into Port B. As stated above, FCCP and DCCD **CANNOT** be used in the same well. Wells should either be injected with only FCCP or DCCD and, subsequently for both, sodium azide.
   1. 24 well: 75 µL per drug, per injection
   2. 96 well: 25 µL per drug, per injection
   3. ***Note: if an injection is programmed, all ports for that injection must be loaded with some liquid (typically K+ if a drug is not needed). For example, if sodium azide injection is programed for Port B all wells being analyzed must have Port B loaded even if the data post injection of Port B is not being utilized
2. Initiate the calibration processed on the XFe96 or XFe24 Seahorse Analyzer assay by clicking on “Review and Run’ —this should take about 20 minutes.
3. Remove the pink adaptor and lid before adding the calibration plate and cartridge into the Seahorse Analyzer. Ensure it is loaded in correct orientation.
4. Wash worms from plates or treatment, count, and dilute to appropriate concentration. Load worms into a fresh cell culture plate cartridge:
   1. 24 well:
      1. Add a total volume of 525 µL of worms in EPA water to each desired well. The number of worms is variable by stage (see table). For control wells, add only 525 µL EPA water with no worms. For the 96 well, these numbers are based on experiments in which readings were consistent but the well sufficiently re-oxygenated with mixing. Our “post-reproductive adult” values are based on day 8 (post-hatch), and may need to be adjusted for much older worms.

| Stage | # per well |
| --- | --- |
| L1 | 2000 (1000 min, 3000 max) |
| L2 | 500 (300 min, 700 max) |
| L3 | 200 (100 min, 300 max) |
| L4 | 75 |
| Young Adult | 50 |
| Post Reproductive Adult | 50 |

- 1. 96 well:
     1. Add a total volume of 100 µL of worms in EPA water to each desired well. The number of worms varies by stage (see table). For control wells, add only 100 µL EPA water with no worms. For the 96 well, these numbers are based on experiments in which readings were consistent but the well sufficiently re-oxygenated with mixing. Our “post-reproductive adult” values are based on day 8 post-hatch, and may need to be adjusted for much older worms.

| Stage | # per well |
| --- | --- |
| L1 | 400 (300 min, 500 max), though difficult to count |
| L2 | untested |
| L3 | ~20 works well; possible range not tested |
| L4 | 15 (10 min, 20 max) |
| Young Adult | 8 (5 min, 10 max) |
| Post Reproductive Adult | 8 (5 min, 10 max) |

- 1. Ensure under microscope that each well has approximately the correct number of worms before proceeding to run

1. On Seahorse, once calibration is complete, click “eject plate” and exchange the calibration plate for the cell culture (worm containing) plate. Ensure plate is loaded in correct orientation.
2. When the machine has accepted the plate, click “proceed”.
3. After the run is completed, image the full plate at a magnification that permits imaging the whole plate and counting worms (in our lab, the Keyence microscope) and manually count the number of worms per well, or quantify with another normalization method
4. Data should be saved on either a personal jump drive or lab drive. Do not store data on the Seahorse computer (in case computer has to be serviced, etc.). The most analysis friendly format is achieved by exporting the results as an excel spreadsheet.
5. Dispose of plates in appropriate chemical waste (solid for plates and sensors, liquid for well contents).

**Data Analysis**

| Parameter | Equation |
| --- | --- |
| Non-mitochondrial OCR | Average rate of 3 last measurements after sodium azide injection |
| Total Basal OCR | Average rate of all but first measurement |
| Mitochondrial Basal OCR | (Total Basal OCR) – (Non-Mitochondrial OCR) |
| Maximal OCR | (average of 3 highest rate measurements after FCCP injection) – (Non-Mitochondrial OCR) |
| Spare capacity | (Maximal OCR) – (Mitochondrial Basal OCR) |
| Spare capacity (fold-change) | (Maximal OCR) / (Total Basal OCR) |
| ATP-linked OCR | (Total Basal OCR) – (average of 2 lowest rate measurements after DCCD injection) |
| ATP-linked as %  (Coupling efficiency) | (ATP-linked OCR) / (Mitochondrial Basal OCR) × 100 |
| Proton leak | (2 lowest measurements after DCCD injection) – (Non-Mitochondrial OCR) |

These are illustrated below.

**
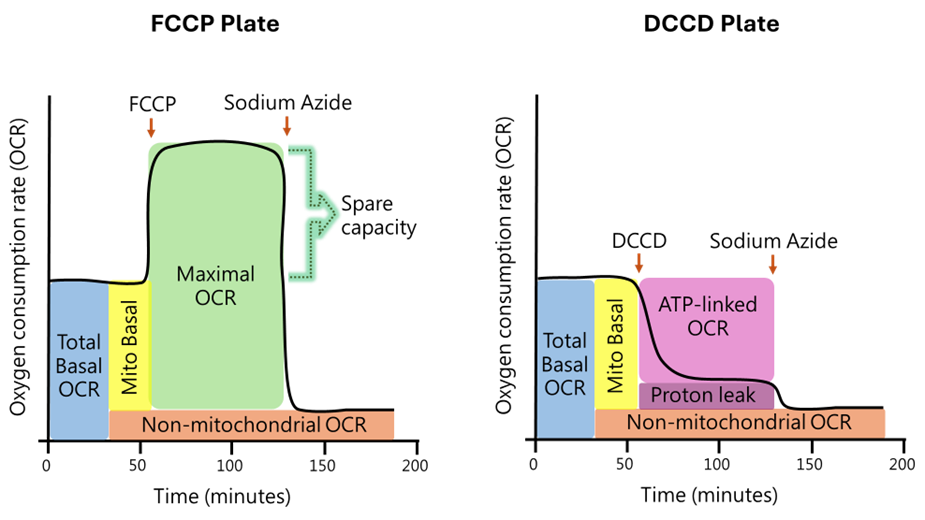
**

**Important!**

**Observe raw OCR values throughout the whole run for each well to check for any issues. Common problems would be:**

1. **Drug injection did not cause any changes in OCR**
   1. if upon injection of FCCP or DCCD no change appears in OCR readings within 3-4 measurements it is likely either the drug was not effective or there was an issue with well oxygenation
2. **Negative OCR levels**
   1. occasionally, wells with insufficient numbers of worms or background wells have read with negative OCR. This is, in part, based on which wells are used as controls for background subtraction. IF many wells appear negative, one of the background wells is likely malfunctioning or contains worms.
3. **Non-linear oxygen consumption caused by oxygen depletion (which can be seen by analyzing the raw oxygen levels)**
   1. When looking at the raw oxygen levels, well oxygen levels should have a baseline that occurs after mixing (oxygenation) and is gradually depleted during the measurement window. During the next mixing window oxygen concentration should return to the same baseline each time. If oxygen concentration fails to return to the same baseline, usually a process that worsens as the run continues, the number of worms per well is too high.
4. **In some cases, you may need to use your judgment to determine if different timepoints (measurements) than those indicated in the Table above should be used for specific calculations.** As one (of many possible) example, if it is clear that 3 post-FCCP measurements include at least one that is much lower than the other two, you may choose to make calculations based on only those two. If unsure, consult with an experienced user.

**Normalization options**

*See also more-detailed lab protocols for each of these, and discussion of their merits in Mello et al., submitted.*

Worm number

Worm number can be based on the number loaded into the wells, but you will get more precise numbers and less well-to-well variability by counting each well afterwards. Image each well or the entire plate via XY stitching and use zoomed-in images to manually count the worms in each well, or use JH’s code to count (not yet available for L1s).

Mitochondrial and Nuclear DNA Copy Number

1. Transfer 6 individual worms using the platinum worm pick or by pipetting (<10 µL) into PCR tubes containing 90µL of 1x worm lysis buffer (30% 3.3x lysis buffer + 5% Proteinase K + 65% Molecular Biology Water) when using L4s and adults – for L1s-L3s use 6 worms in 60 µL
2. Freeze them in the -80°C freezer (they can be stored there until further use)
3. After at least 10 minutes, thaw the samples, vortex and spin them briefly
4. Place them in a thermal cycler to heat to 65 °C for 1 hour, then 95 °C for 15 minutes, then hold at 8 °C. 5 minutes into the cycle, remove tubes, vortex and spin briefly. The worm lysate will be used as template DNA.
5. Thaw an aliquot of the mtDNA copy number standard curve plasmid (*nd-1* gene) (10,000,000 copies/µL)
6. Dilute to 1,000,000 copies/µL, and serial dilute 1:1 down to 1953 copies/µL (10 total dilutions) and include a no template control. This standard curve can be adjusted as needed based on age of the worm
7. Thaw an aliquot of the nDNA copy number standard curve plasmid (*cox-4* gene) (6,500,000 copies/µL)
8. Dilute to 12800 copies/µL, and serial dilute 1:1 down to 25 copies/µL (10 total dilutions) and include a no template control. This standard curve can be adjusted as needed based on age of the worm
9. Add 2µL of the worm lysate, 0.1µL of each forward and reverse primer (100µM), 12.5µL Power SYBR Green PCR Master Mix), and 10.3µL of water in each well of the 96-well PCR plate (*do this both for nDNA –using the cox-4 gene primer set– and mtDNA – using the nd-1 gene primer set*)
10. Amplify the target DNA using the Analytik Jena qTOWER^3^ at 50 °C for 2 minutes, 95°C for 10 minutes, 40 cycles of 95°C for 15 seconds
11. In the software, select the option to calculate a dissociation curve for each sample to ensure the presence of a single product (Rooney et al., 2015 <https://pubmed.ncbi.nlm.nih.gov/25308485/>, Leuthner et al., 2021 <https://pubmed.ncbi.nlm.nih.gov/34096001/>)

Size

1. After inserting the plate and cartridge into the Seashorse Analyzer, re-transfer the remaining worms from 15ml centrifuge tube back to the seeded K-agar NO PEPTONE plate using a Pasteur Pipette or 0.1% X-triton-100 rinsed tips (*if they are suspended in a large volume, re-pellet them before transfer, otherwise it will take a long time for the plate to dry*)
2. Let the plate dry
3. Turn on the Keyence BZ-X710 microscope
4. Load the microscope with plate and identify its boundaries on the software (*you can load a pre-saved settings file for the plate you will be using, but you’d just have to adjust the focus each time*)
5. Initiate a Plate Scan
6. Save the pictures in working folder and flash drive
7. Upload the pictures on Worm Sizer (Moore et al., 2013 <https://pubmed.ncbi.nlm.nih.gov/23451165/>) for analysis
8. Follow the worm sizer protocol to measure worm length/area/volume

Total Protein Content

1. Transfer at least two samples of worms *(~2000 if L1-L2 and ~1000 if L3-adult - these numbers are not strict, as long as the number of worms is known and somewhat close to these values*) and freeze samples in the -80°C freezer until further use
2. Thaw worms, centrifuge at 2200g for 2 minutes
3. Aspirate supernatant
4. Add 100-250 µL of 10% SDS to each sample
5. Ultrasonication options:
   1. Place samples in bath sonicator in chilled water (*add ice cubes to keep water chilled, but be careful to not let it become too cold, as the 10% SDS solution may freeze*) and observe samples under light microscope to complete nematode lysis (*depending on the stage, this could take from 40 minutes to 2 hours*);
   2. Use Model 3000 Ultrasonic Homogenizer (BioLogics, Inc.) located in the equipment lab and sonicate for 2 cycles of 30s with the lowest amplitude setting
6. Follow instructions in Thermo Fischer BCA assay to extract protein
7. Prepare standard curve containing known concentrations of bovine serum albumin (1000, 750, 500, 250, 125, 125, 0 ug/ml) in the presence of 10% SDS

**Lab Notes and Observations**:

Although rare, there may be a time when your seahorse file does not save. This could be from a power outage or cosmic anomaly (who really knows what happens when nobody is in the lab). Seahorse automatically saves a backup file on the computer; you just have to find it. The actual pathway I haven’t quite figured out yet but if you search My Computer (all files) for the “.asyr” extension it will find the backup if the seahorse run finished before the anomaly. I do not know what exactly happens when the glitch happens mid-run. - CMB

For both the 24 and 96 well, normalization can be added directly into the seahorse software so the calculations do not need to be completed in excel 😊 -KM

There is often a big dip in OCR the first read after injection. This is likely because the injection added enough oxygen to depress the apparent rate of oxygen consumption in that first reading.

In our original publications for Seahorse in worms, we described the rate of oxygen consumption in units of pmol/min/mg protein; they should instead be listed as nmol/min/mg protein. This has been corrected in the Luz et al., 2015 PLoS ONE paper, but not the Current Protocols paper (we tried, but the journal has no mechanism to allow this).

The 96-well Seahorse has installed the plate pad for round-bottom plates. Our plates are flat bottom, so the software likes to reject the barcodes for our plates, easily solved by taking a barcode from a round bottom plate and using that number instead. – JH

What to do with negative non-mitochondrial OCR numbers: In some cases, OCR after azide addition may be negative, indicating on the face of it an increase in oxygen concentration over time. This of course is not biologically possible (worms do not photosynthesize!), and presents technical challenges, including for calculation of basal mitochondrial OCR and maximal OCR (since these calculations include subtraction of non-mitochondrial OCR). The ideal option is to repeat the experiment. A second option is to define non-mitochondrial OCR measurements that are negative as zero. -JNM

DMSO: Tony Luz (2015 PLOS ONE paper) found that 2% DMSO with DCCD led to a slight increase in OCR (not with FCCP, probably due to the length of reads), and so recommended using 1%, which did not affect OCR with DCCD. However, he also reported that concentrations over 20 μM DCCD were not soluble at 1% DMSO, and we now recommend 40 or 80, and so recommend 2% DMSO in most cases. -JNM

Koopman et al. (<https://pubmed.ncbi.nlm.nih.gov/27583642/> Fig. S2) and Haroon and Vermulst (<https://pubmed.ncbi.nlm.nih.gov/33654802/>) report minimal effect of bacteria on OCR.

**Recipes**:

K-medium:

### 1 L 2 L

KCl 2.36 g 4.72 g

NaCl 3 g 6 g

dH_2_O 1000 ml 2000 ml

Combine in flask, cover with foil, autoclave (with adequate number of 500ml squeeze bottles).

Reference: Williams, P. L.; Dusenbery, D. B. Using the Nematode Caenorhabditis-Elegans to Predict Mammalian Acute Lethality to Metallic Salts. Toxicol. Ind. Health 1988, 4 (4), 469−478.

EPA Water:

In 1 L ddH_2_O

60 mg anhydrous MgSO_4_ **dissolve this first!**

96 mg NaHCO_3_

60 mg CaSO_4_*2H_2_O

4 mg KCl

Reference: EPA, Methods for measuring the acute toxicity of eluents and receiving waters to freshwater and marine organisms. 2002.
