# Supplementary figures and images for: Comprehensive characterization of mitochondrial bioenergetics at different larval stages reveals novel insights about the developmental metabolism of Caenorhabditis elegans
Source: PLoS One. 2024 Nov 26;19(11):e0306849. doi: 10.1371/journal.pone.0306849 (PMC11593755; doi:10.1371/journal.pone.0306849)

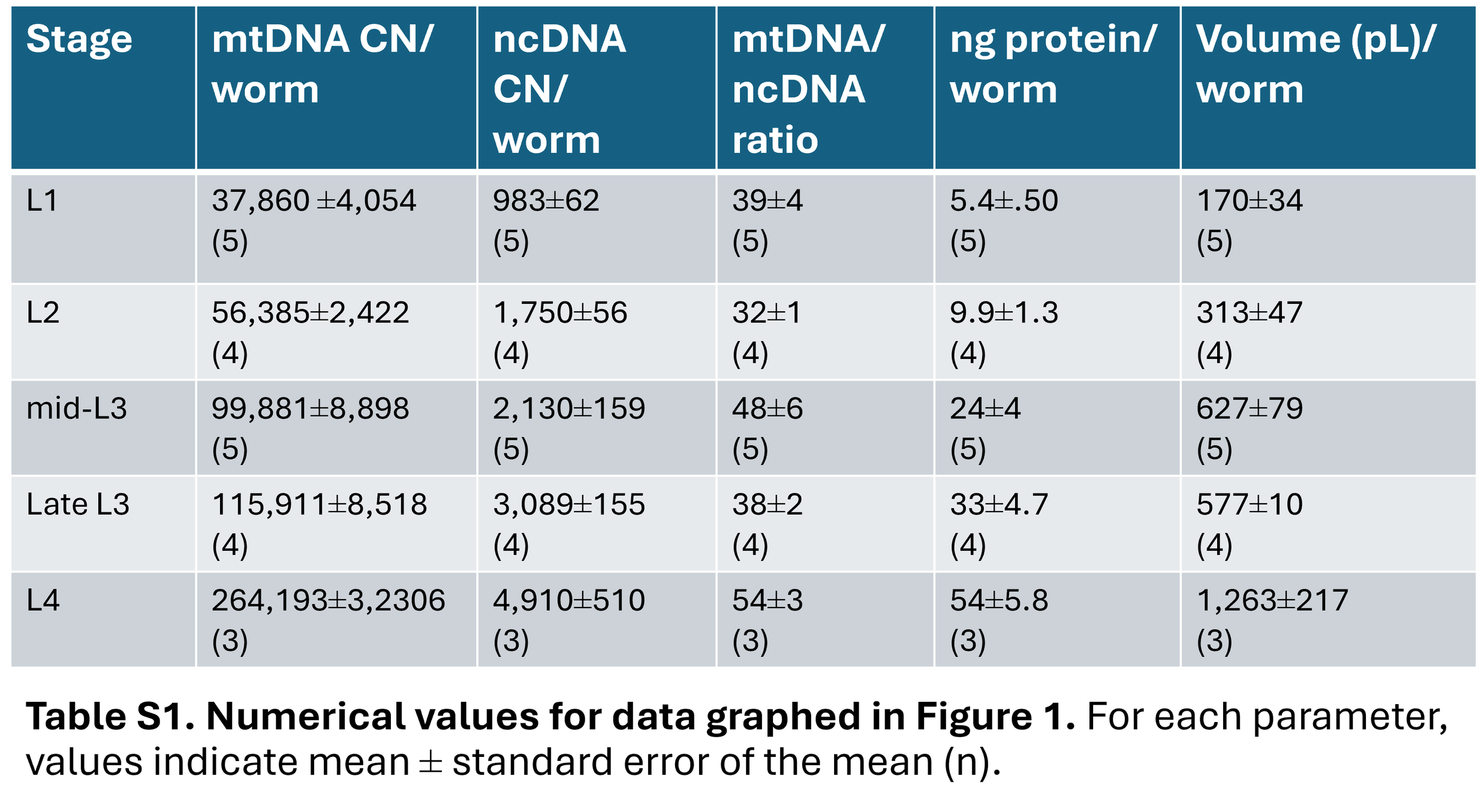

Supplement: S1 Table — (TIF) [file pone.0306849.s003.tif]

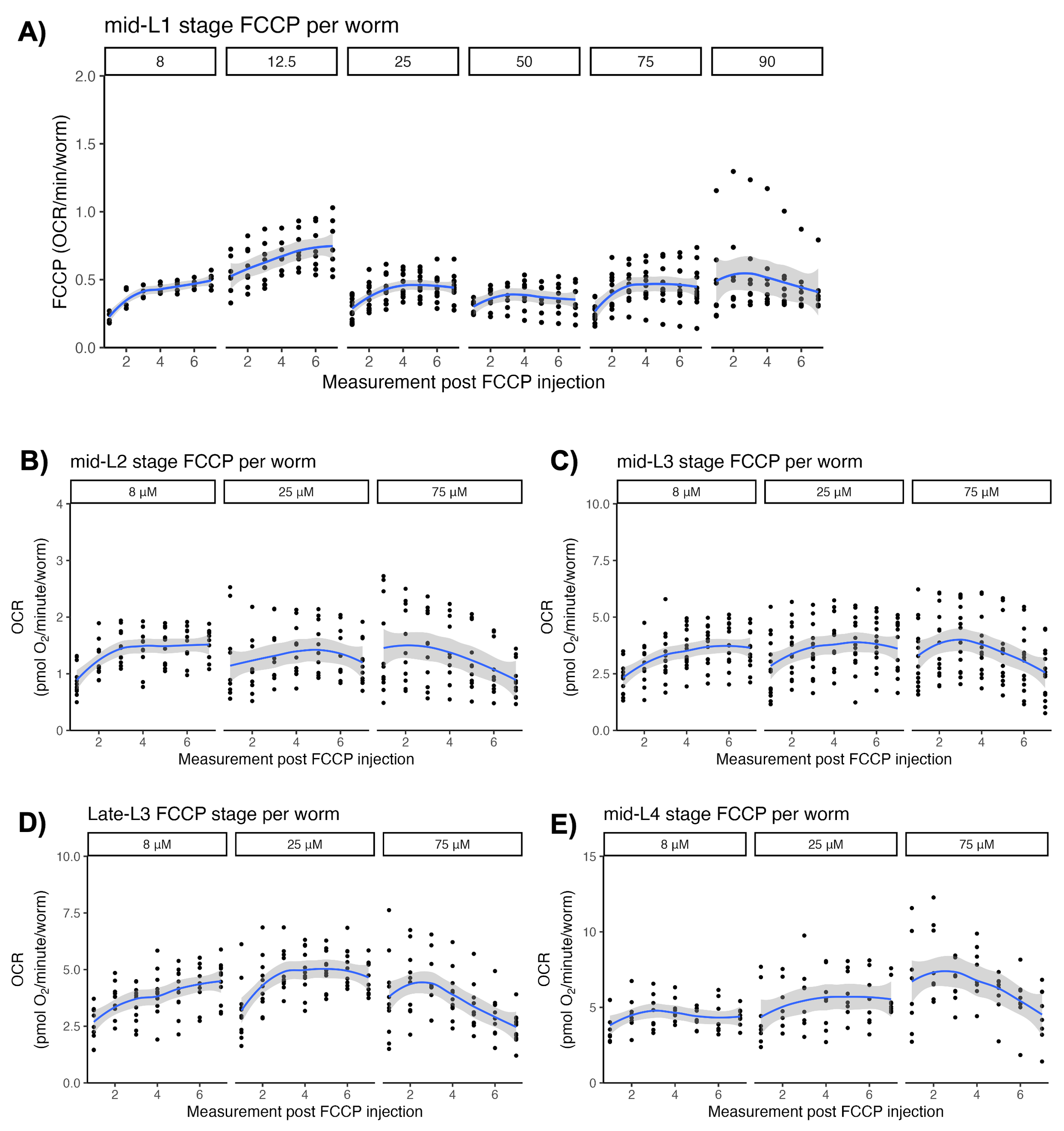

Supplement: S1 Fig — S2 Fig shows oxygen consumption rates at the L1 (panel A), L2 (panel B), mid-L3 (panel C), late L3 (panel D), and L4 (panel E) stages at different timepoints after the injection of different concentrations of FCCP, normalized per worm. Blue line represents local polynomial regression fitting of the data for visualization with 95% confidence interval using the geom_smooth function in ggplot2 package in R version 4.2.1. Figures A-E represent data across 1–4 biological replicates with 3–6 technical replicates (L1–1–3 biological reps with 4–5 technical replicates; L2 –three biological reps with 3–5 technical replicates; L3 –four biological reps with 3–5 technical replicates; Late-L3- three biological reps 3–5 technical replicates; L4 –two biological reps with 3–5 technical replicates). (TIF) [file pone.0306849.s004.tif]

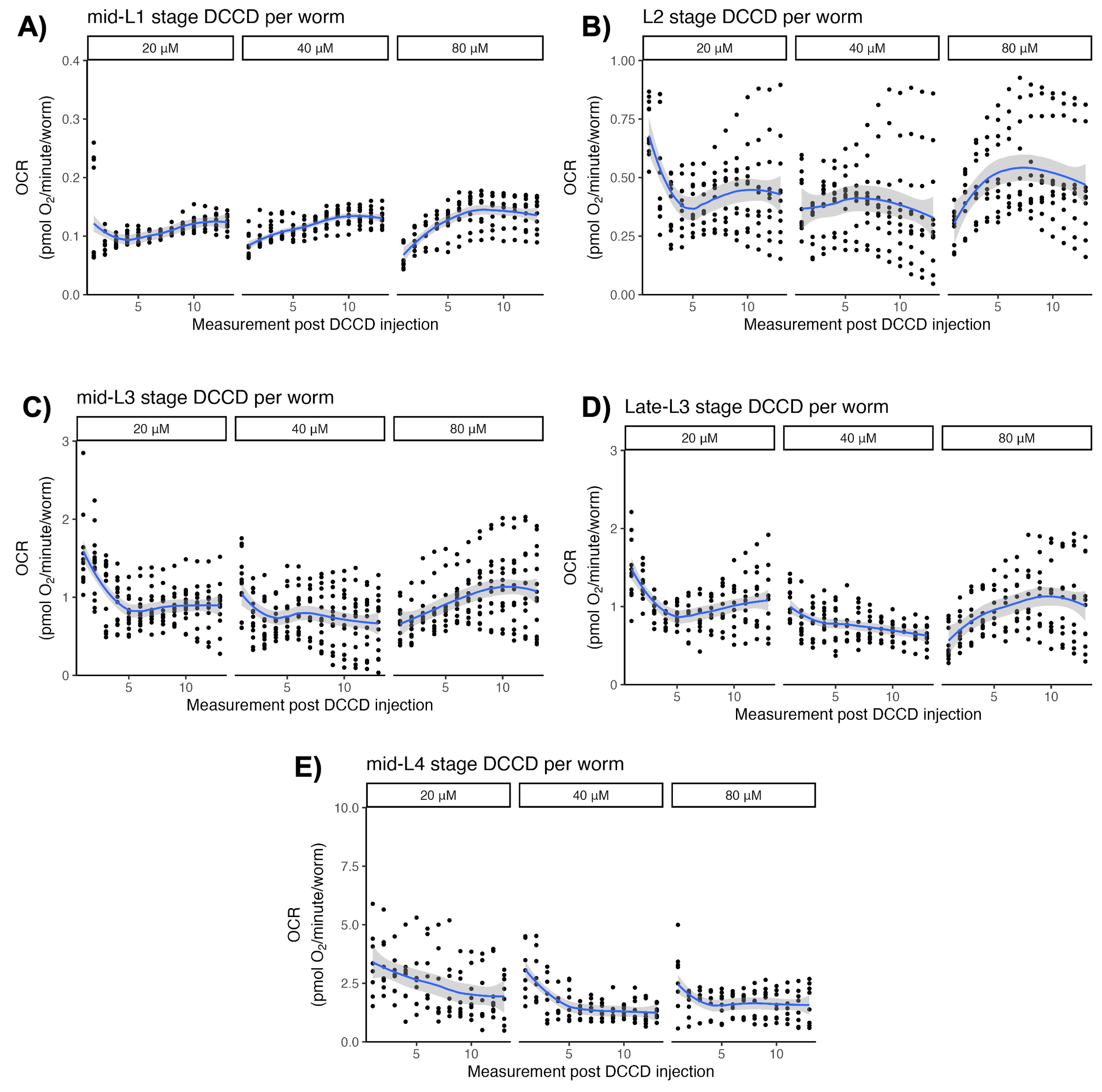

Supplement: S2 Fig — S3 Fig shows oxygen consumption rates at the L1 (panel A), L2 (panel B), mid-L3 (panel C), late L3 (panel D), and L4 (panel E) stages at different timepoints after the injection of different concentrations of DCCD, normalized per worm. Blue line represents local polynomial regression fitting of the data for visualization with 95% confidence interval using the geom_smooth function in ggplot2 package in R version 4.2.1. Figures A-E represent data across 2–4 biological replicates with 3–6 technical replicates (L1 –two biological reps with 4–6 technical reps; L2 –three biological reps with 3–5 technical reps; L3 –four biological reps with 3–5 technical reps; Late-L3- three biological reps with 3–5 technical reps; L4 –two biological reps with 3–5 technical reps). (TIF) [file pone.0306849.s005.tif]

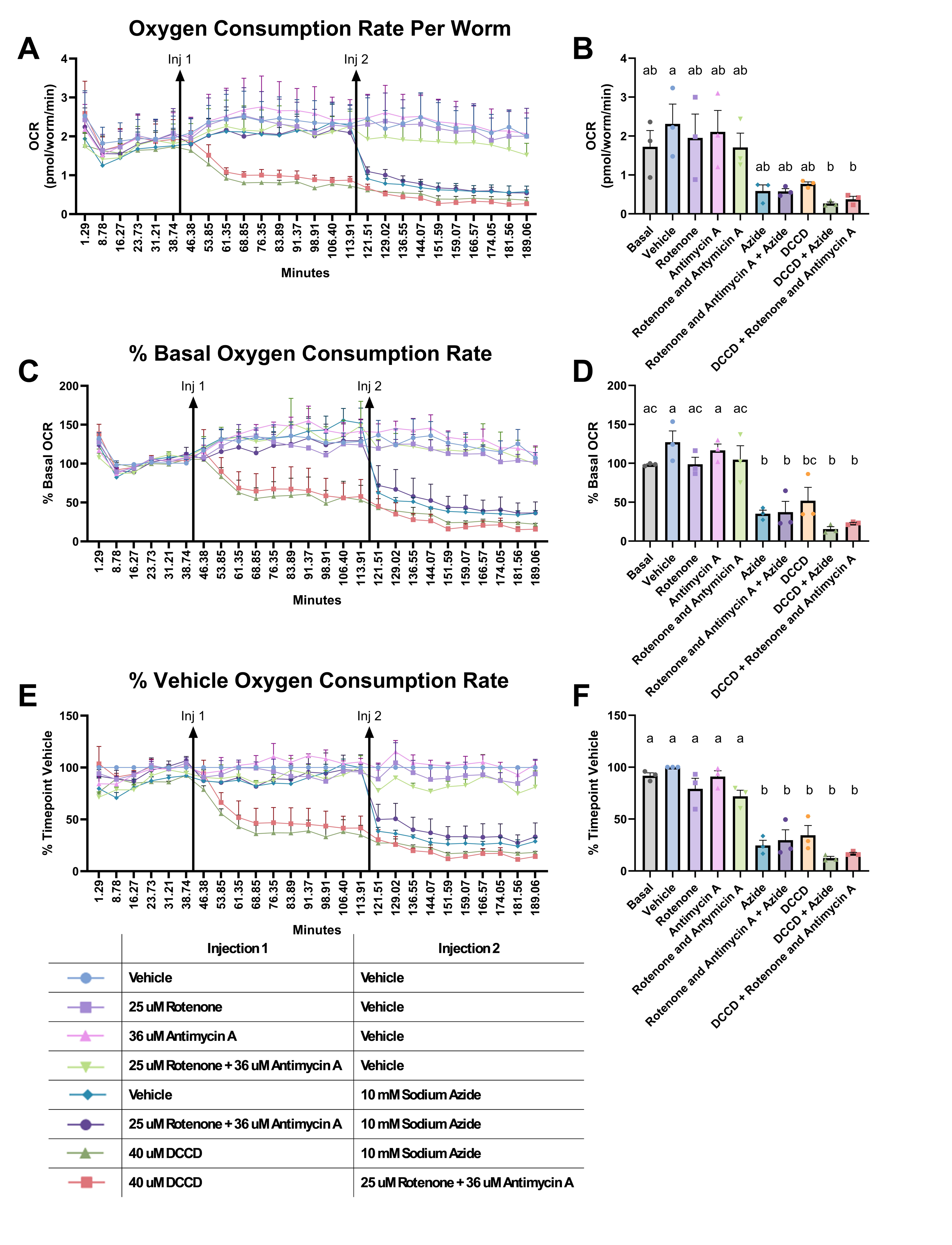

Supplement: S3 Fig — For comparison to 10 mM sodium azide, maximum soluble doses of rotenone (25 μM) and antimycin A (36 μM) in 1% DMSO were examined alone, in combination, and after injection of 40 μM DCCD. Three biological replicates were performed utilizing L4 worms with 5–11 technical replicates per group. First, OCR per worm is shown throughout the course of the assay (A) and the calculated OCR corresponding to each treatment (B). To improve visual clarity and comparability, the results are also shown normalized to the treatment specific basal measurements occurring prior to the first injection (C, D), and the timepoint matched vehicle control (E, F). Statistical analysis was performed utilizing GraphPad Prism 10.2.3. A two-way ANOVA was used for each analysis, followed by Sidak’s post-hoc for multiple comparisons with p<0.05 as the cutoff for statistical significance. Comparisons are displayed by letter in which any treatment possessing the same letter is not statistically significantly different and only treatments that have no identical letters are significantly different. (TIF) [file pone.0306849.s006.tif]

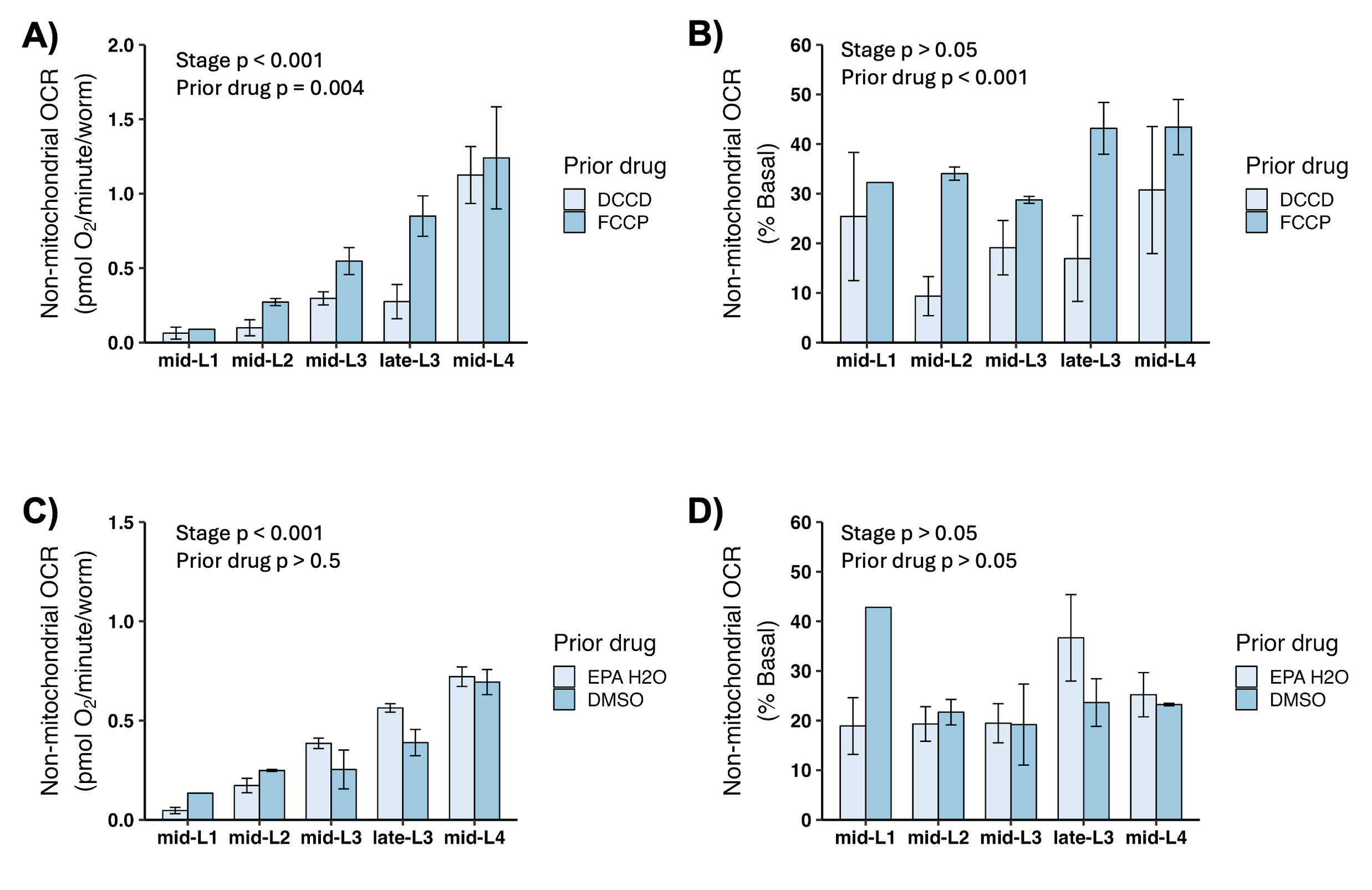

Supplement: S4 Fig — Non-mitochondrial OCR normalized per worm and percent basal after injection with FCCP and DCCD. n = 1–4 biological replicates, p-values from two-way ANOVA. (TIF) [file pone.0306849.s007.tif]
