# Supplementary material for: Comprehensive characterization of mitochondrial bioenergetics at different larval stages reveals novel insights about the developmental metabolism of Caenorhabditis elegans
Source: PLoS One. 2024 Nov 26;19(11):e0306849. doi: 10.1371/journal.pone.0306849 (PMC11593755; doi:10.1371/journal.pone.0306849)
Supplement: Attachment — Submitted filename: Response to Reviewers_9_20.docx [file pone.0306849.s009.docx]

Dear Editors,

Thank you for the opportunity to submit a revised draft of our manuscript “*Comprehensive characterization of mitochondrial bioenergetics at different larval stages reveals novel insights about the developmental metabolism of Caenorhabditis elegans.*” Please see below, in blue text, our responses to each of the reviewer comments. All page numbers refer to the clean version of the revised manuscript.

**Reviewer #1:**

The requested modifications have been scrupulously carried out.

These requested modifications were, in the majority of cases, necessary to allow greater clarity in the tools used to carry out this study. Corrections to the material and methods section have been carefully drafted to increase the clarity of the technique used in this manuscript.

The changes requested in the “Supporting info section” have been made, as have the minor modifications requested.

I recommend publication of this manuscript.

(No concerns to address)

**Reviewer #2:**

Thanks the authors for your thoughtful responses. All of my comments have been fully addressed. The updated Figure 1 clearly demonstrates how the measurements were done.

(No concerns to address)

**Reviewer #3:**

**But the authors are probably joking about measuring a metabolic profile with their method!**
We are not sure we understand. We do not mention a “metabolic profile.” The only time we use the word profile” in the manuscript is in this sentence: “To establish the profile of *C. elegans* mitochondrial bioenergetics throughout different life stages we stage-synchronized wild-type (N2) nematodes (Figure 2A, 2B) and measured OCR levels with and without different drugs (electron transport chain inhibitors and a mitochondrial uncoupler).” In this case, “profile” simply refers to “characterization.”

**Another mistake. Sodium azide is an inhibitor of ATP synthase. Are the authors aware of this?**
We are not clear what the reviewer describes as a mistake. We are aware that sodium azide is (also) an inhibitor of ATP Synthase, and so state in the manuscript (Lines 438-44): “Non-mitochondrial OCR is measured by inhibiting all oxidative phosphorylation using sodium azide, a potent complex IV/V inhibitor.”

**However, the oxygen consumption profiles with the data obtained from their experiments are not presented. Figure 1A is a graphical construction!!! This is enough to consider the results not tenable.** Yes, Figure 1A is a graphical illustration of how values were calculated and terms defined, as was requested by Reviewer 2. We consider (and note that Reviewers 1 and 2 appear to agree) that our Results are clearly shown, and Conclusions fully supported, by the Figures and data presented. As stated in our previous Response to Reviewers (9/10), “all the OCR curves (but not full profiles) are presented within the supplementary files.” Showing full OCR profiles for every experiment would require dozens of additional Figures, and would add almost no value beyond that provided by the raw data, all of which is provided in Supplemental (S1 File).
